# The neuropeptide Y Y1 receptor knockdown modulates activator protein 1-involved feeding behavior in amphetamine-treated rats

**DOI:** 10.1186/1756-6606-6-46

**Published:** 2013-11-13

**Authors:** Yih-Shou Hsieh, Pei-Ni Chen, Ching-Han Yu, Jiuan-Miaw Liao, Dong-Yih Kuo

**Affiliations:** 1 Institute of Biochemistry and Biotechnology, Chung Shan Medical University and Chung Shan Medical University Hospital, Taichung City 40201, Taiwan; 2 Department of Physiology, Chung Shan Medical University and Chung Shan Medical University Hospital, Taichung City 40201, Taiwan

**Keywords:** NPY-Y1 receptor, c-Fos, c-Jun, AP-1, Appetite, Hypothalamus

## Abstract

**Background:**

Hypothalamic neuropeptide Y (NPY) and two immediate early genes, *c-fos* and *c-jun*, have been found to be involved in regulating the appetite-suppressing effect of amphetamine (AMPH). The present study investigated whether cerebral catecholamine (CA) might regulate NPY and POMC expression and whether NPY Y1 receptor (Y1R) participated in activator protein-1 (AP-1)–mediated feeding.

**Methods:**

Rats were given AMPH daily for 4 days. Changes in the expression of NPY, Y1R, c-Fos, c-Jun, and AP-1 were assessed and compared.

**Results:**

Decreased CA could modulate NPY and melanocortin receptor 4 (MC4R) expressions. NPY and food intake decreased the most on Day 2, but Y1R, c-Fos, and c-Jun increased by approximately 350%, 280%, and 300%, respectively, on Day 2. Similarly, AP-1/DNA binding activity was increased by about 180% on Day 2. The expression patterns in Y1R, c-Fos, c-Jun, and AP-1/DNA binding were opposite to those in NPY during AMPH treatment. Y1R knockdown was found to modulate the opposite regulation between NPY and AP-1, revealing an involvement of Y1R in regulating NPY/AP-1–mediated feeding.

**Conclusions:**

These results point to a molecular mechanism of CA/NPY/Y1R/AP-1 signaling in the control of AMPH-mediated anorexia and may advance the medical research of anorectic and anti-obesity drugs.

## Background

Amphetamine (AMPH) is a well-known psychostimulant. Although AMPH has a neurotoxic effect because of the production of free radicals and oxidative damage in the brain [1,2], AMPH can be clinically applied to improve some nervous disorders, such as childhood attention-deficit/hyperactivity disorder [3], multiple sclerosis patients with memory impairment [4], stroke or brain injury [5], and Alzheimer’s disease [6]. Regarding the effect on appetite, AMPH recently served as a prototype for the research on anorectic drugs for the development of subsequent anti-obesity drugs [7,8]. Therefore, the mechanisms behind the anorectic, psychomotor, and neurotoxic effects of AMPH have been investigated extensively.

The mechanism underlying the appetite-suppressing effect of AMPH is relevant to the central release of catecholamine (CA), which may act on hypothalamic NPY-containing neurons to suppress appetite [9]. Both neuropeptide Y (NPY) and proopiomelanocortin (POMC) are involved in regulating AMPH-induced anorexia [10,11]. However, it is still unknown whether cerebral CA is involved in this regulation, we hypothesized that decreased CA could simultaneously modulate NPY and POMC expression in AMPH-treated rats.

Hypothalamic NPY is a highly conserved neuropeptide that contributes controlling daily feeding behavior, energy homeostasis [12,13], stress [14], and anxiety [15]. Thus, NPY and its receptors have been implicated in various biological functions and neuronal disorders, such as epilepsy, obesity, and anxiety. NPY acts on at least five receptors, including the Y1, Y2, Y4, Y5, and y6 subtypes, which are class I G-protein–coupled receptors [16]. Of these subtypes, the Y1 receptor (Y1R) and the Y5 receptor (Y5R) have been suggested to mediate the effect of NPY-mediated feeding [17,18]. Studies have shown that both Y1R and Y5R knockout mice have higher body weight, increased food intake, and greater adipose deposition [19,20]. Recently, reports revealed that hypothalamic Y1R, but not Y5R, was involved in the regulation of AMPH-induced anorexia [21] or phenylpropanolamine (PPA)-induced anorexia [22], revealing a major role of Y1R in CA-mediated anorexia.

In the nervous system, there are two immediate early genes (IEG), *c-fos* and *c-jun*, that can be induced by extracellular signaling, including hormones, neurotransmitters, and drugs of abuse [23]. The cascade of c-Jun and c-Fos signaling can be activated by AMPH [24-26]. The activator protein-1 (AP-1) binding protein, which is formed as a Fos/Jun heterodimer or a Jun/Jun homodimer of IEG, is a representative transcriptional factor that is activated in response to signal transduction cascades [27]. Thus, AP-1 activity is increased in several brain regions after methAMPH treatment [28], and AP-1 DNA binding activity is associated with enhanced motor behavior in AMPH-treated rats [29]. Previously, we reported that c-Fos/c-Jun participated in NPY-mediated appetite regulation in AMPH-treated rats [26]. However, it is still unclear whether Y1R and AP-1 signaling participated in the regulation of the NPY-mediated feeding. We hypothesized that the activation of NPY/Y1R/AP-1 signaling might be involved in the control of AMPH-induced anorexia.

## Methods

### Animal treatments

Male rats of the Wistar strain, with a weight of 200 ~ 300 g, were obtained from the National Laboratory Animal Center. They were housed individually in cages, maintained at a temperature of 22 ± 2°C, in a room with a 12-hour light-dark cycle (light on at 6:00 AM), and habituated to frequent handling. The administration of drugs and the checking of food intake were performed every day at the beginning of the dark phase (6:00 PM). All procedures were carried out in accordance with the Guide for the Care and Use of Laboratory Animals as adopted by the National Institutes of Health. This study has been approved and reviewed by the National Science Council, Taiwan, ROC.

### Experimental procedures

To examine the effect of daily AMPH (*d*-amphatamine, a sulphate salt dissolved in saline) on feeding behavior, rats (*N* = 8 for each group) were injected intraperitoneally (i.p.) with various doses of AMPH (0, 2 or 4 mg/kg) once a day for 4 days. The first time AMPH was given was at the end of Day 0 (at 6:00 pm), which was regarded as the beginning of Day 1. The intake data were calculated as the total amount of food during the previous day. To examine the effect of endogenous CA on daily AMPH anorexia, α-methyl-para-tyrosine (AMPT) was given prior to the treatment of 4-mg/kg AMPH once a day for 4 days. In a previous study, AMPT, an inhibitor of tyrosine hydroxylase, was given with the dose of 40 mg/kg (i.p.) into rats twice a day at 6 and 2 h prior to AMPH administration in order to inhibit CA synthesis in the brain [30]. AMPT treatment can efficiently decrease CA content in the brain as described in our previous report [9]. The treatment of AMPT alone has no significant effect on feeding behavior.

To determine the effect of pretreatment with Y1R antisense oligodeoxynucleotide (ODN) on anorectic response of AMPH, rats (*N* = 8 for each group) were given intracerebroventricularly (i.c.v.) with missense (control group) or antisense (20 μg in a 10-μl vehicle) once a day at 1 h before AMPH (4 mg/kg; i.p.) for 4 days. Before AMPH treatment, rats were given with similar dose of missense (or antisense) daily for 2-3 days until the response of feeding behavior was changed slightly in antisense group. This was due to the fact that either continuous or repeated injections of antisense might be necessary to maximize behavioral effect and especially to block the synthesis of constitutively active gene product [31,32].

To assess the effect of daily AMPH on hypothalamic NPY, Y1R, c-Fos, and c-Jun, levels, rats (*N* = 8 each group) were given with AMPH (0 or 2 mg/kg; i.p.) once a day for 1, 2, 3 or 4 days depending on the group of rats. Rats were divided into 5 groups (*N* = 8 for each group) according to the day they were to be sacrificed. Rats received AMPH at 40 min prior to being anesthetized (pentobarbital, 30 mg/kg, i.p.) and decapitated to remove hypothalamus from the brain immediately, which was then subjected to determinations of protein levels or stored at –80°C until further use.

To determine the effect of AMPH on AP-1/DNA binding activity, rats were given with the AMPH (4 mg/kg; i.p.; *N* = 6-8 each group) daily for 4 days at the beginning of dark phase (at 6:00 PM). At 40 min after daily AMPH treatment, the hypothalamus was removed daily to determine AP-1/DNA binding activity by a technique of chromatin immunoprecipitation (ChIP) assay.

To examine the effect of Y1R antisense (or missense) on NPY, c-Fos, c-Jun, and Y1R levels in AMPH-treated rats, rats (*N* = 8 for each group) were infused daily with antisense or missense (20 μg in a 10-μl vehicle; i.c.v.) at 1 h before daily treatment with 2 mg/kg AMPH for 4 days. Before AMPH treatment, rats were infused with similar dose of antisense (or missense) daily for 2-3 days. At 40 min after antisense (missense) and/or AMPH treatment, rat’s hypothalamus was removed for the determination of protein levels.

To determine the effect of Y1R antagonist on AMPH-induced anorexia and on the changes of hypothalamic NPY, c-Fos, and c-Jun levels during a 24-h testing period, rats (*N* = 8 for each group) were pretreated with BIBP-3226 at 30 min before 2 mg/kg AMPH treatment. BIBP-3226 is developed as an Y1R antagonist, which is known not to have any effect at the Y2, Y4, and Y5 receptors [33] and can significantly reduce NPY-induced feeding [34]. We therefore studied the effect of BIBP-3226 (80 nmole, i.c.v.; MW 473.6) on AMPH-induced effects. Rats received BIBP and/or AMPH at 40 min prior to the removal of hypothalamus. The BIBP-3226 is dissolved in artificial cerebrospinal fluid (aCSF) solution containing 140 mM NaCl, 3.35 mM KCl, 1.15 mM MgCl_2_, 1.26 mM CaCl_2_, 1.2 mM Na_2_HPO_4_, 0.3 mM NaH_2_PO_4_, pH 7.4.

### Lateral ventricular cannulation

A surgery of rat was performed under anesthesia with pentobarbital (30 mg/kg; i.p.) using stereotaxic apparatus (Kopf Model 900, Tujunga, CA, USA). The target of cannulation was close to the junction between the right lateral ventricle and the third ventricle (coordinates: 0.8 mm posterior to Bregma, 1.5 mm from the midline, and 3.5 ~ 4.0 mm below the dura) [35]. A 23-g stainless steel guide cannula was implanted and secured to the skull using stainless-steel screws and dental cement. A correct placement was confirmed by observing a transient and rapid inflow of vehicle in PE tube connected with a 28-g injector cannula. The cannula was then occluded with a 28-g stylet. For ICV infusion of antisense, the stylet was replaced with a 28-g injector cannula extending 0.5 mm below the tip of guide cannula. Behavioral testing began at 1 week after the surgery. For all experiments, verification of cannula placement was done by the administration of angiotensin II (100 ng/rat) and by the histological checking. Angiotensin II reliably induced water drinking in non-deprived rats when administered into the ventricles [36]. Only data from rats drinking more than 10 ml within 30 min were included in this study.

### ICV injection of Y1R antisense

Y1R antisense ODN was administered as described previously [37] except for the change of dosage and the modification of sequence at both ends. Briefly, 40 μg of YlR-antisense or control (missense) dissolved in 20 μl sterile vehicle was injected twice daily (20:00 PM and 8:00 AM) for three consecutive days, since this treatment has been shown to selectively reduce Y1R density, with no effects of the sense ODN [37]. The base sequences were: antisense, 5′-GGAGAACAGAGTTGAATT-3′ and missense, 5′-AATTCAACTCTGTTCTCC-3′. We used ODNs that were phosphorothioate-modified (S-ODN) only on the three terminal bases of both the 5′ and 3′ ends, because these S-ODNs had been shown to produce sequence-specific effects without detectable toxicity in brain region and was regarded as a well-established agent in several vertebrate systems [32,38]. Moreover, we selected a dose of 20 μg of antisense S-ODN because previous studies had shown that i.c.v. injections of this amount of antisense optimally inhibited the expression of genes and the activity of feeding behavior [39,40]. Both antisense and missense S-ODN were dissolved in aCSF solution.

### Western blotting

Protein samples extracted from hypothalamus tissue were separated in a 12.5% polyacrylamide gel, transferred onto a nitrocellulose membrane and then incubated separately with specific antibodies against NPY, Y1R, c-Fos, c-Jun, and β-actin. The β-actin was used as an internal standard of protein. After incubation with horseradish peroxidase goat anti-rabbit IgG, the color signal was developed by 4-chloro-1-napthol/3,3′-diaminobenzidine, 0.9% (w/v) NaCl in Tris-HCl. Relative photographic density was quantified by scanning the photographic negative film on a Gel Documentation and Analysis System (AlphaImager 2000, Alpha Innotech Corporation, San Leandro, CA, USA).

### Chromatin immunoprecipitation (ChIP) assay

ChIP analysis was performed as described previously [41]. Chromatin isolation and ChIP assay were performed by using the EZ-ChIP chromatin immunoprecipitation kit (Millipore, Bedford, MA, USA) according to the manufacturer’s instructions. Briefly, after fixation of hypothalamus tissue with 1% formaldehyde, each soluble chromatin was digested and isolated using EZ-Zyme lysis buffer and EZ-Zyme enzymatic cocktail, 4 × 10^6^ cells that were isolated from chopped mouse brain tissue and then 2.5 mol/L glycine solution was added to stop the cross-linking reaction. The chromatin fraction was diluted 10-fold with ChIP dilution buffer and precleared with salmon sperm DNA in a protein G agarose. The precleared chromatin solution was divided and used in immunoprecipitation assays with anti-c-Jun, anti-c-Fos and anti-rabbit IgG antibodies. Following multiple washes, the antibody-protein-DNA complex was eluted from beads. After reversal cross-link incubation, protein and RNA were removed by proteinase K and RNase A. Purified DNA was subjected to polymerase chain reaction (PCR) with primers specific for AP-1-binding sites upstream of the transcriptional start site. The sequences of the PCR primers used are as follows: AP-1, 5′-CCT AAG GCA TAG AGC AAT GAC-3′ (sense) and 5′-GGT GAG AAA CAT GAC TAG GTG-3′ (antisense). Extracted DNA (2 μl) was used for 45 cycles of amplification in 50 μl of reaction mixture under the following conditions: 95°C for 30s, 58°C for 60s, and 72°C for 30s. The PCR products were analyzed by 2% agarose gel electrophoresis [42].

### Drugs, chemicals and reagents

Chow (LabDiet) was purchased from PMI Nutrition International (Brentwood, MO, USA). AMPH, AMPT, BIBP-3226, Tris-HCl solution, angiotensin II, ethidium bromide were purchased from Sigma-Aldrich (St. Louis, MO, USA); antibody against NPY and c-Fos were purchased from Santa Cruz Biotechnology (Santa Cruz, CA, USA), those against AP-1 and c-Jun antibodies were purchased from Cell Signaling Technology, (Beverly, MA, USA), while that against β-actin was purchased from Gibco BRL, Life Technologies, Inc., (Rockville, MD, USA). Anti-NPY1R polyclonal antibodies were purchased from Novus Biologicals, LLC (Littleton, CO, USA). TRIZOL reagent (Life Technologies, Inc., Grand Island, USA) was used in tissue homogenization. Antisense Y1R was synthesized by Proligo Pty Ltd (Singapore).

### Statistical analysis

Data were presented as the mean ± SEM. Two-way or one-way ANOVA followed by Dunnett’s test was used to detect significances among groups. P < 0.05 was considered to be statistically significant.

## Results

### The effect of AMPT pretreatment on AMPH-induced appetite suppression and NPY/MC4R expression

**Figure 1:**
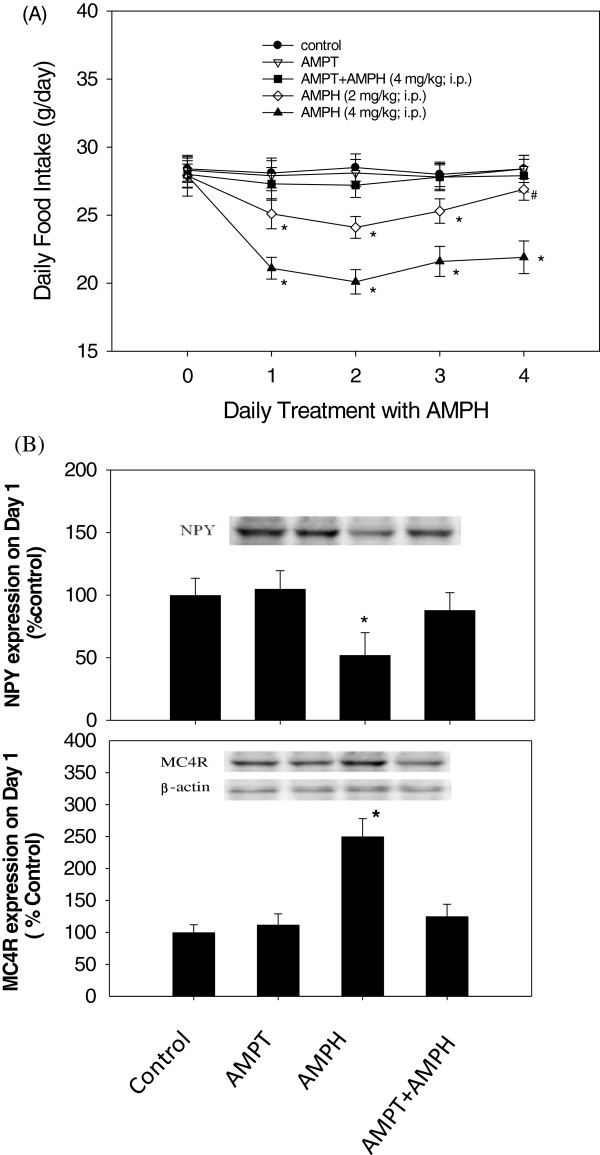
**Effect of AMPT and/or AMPH administration on feeding behavior and hypothalamis NPY and MC4R expression. (A)** The effect of AMPT and/or AMPH administration on food intake over a 4-day period. The first injection of AMPH (0, 2 and 4 mg/kg; i.p.) was conducted at the end of Day 0. AMPT (40 mg/kg; i.p.) was administered twice a day prior to daily AMPH (4 mg/kg). Each point represents the mean ± SEM of 8 rats. *P < 0.05 vs. the control group. ^#^P < 0.05 vs. the AMPH (2 mg/kg)-treated group on Day 2. **(B)** The effect of AMPT, AMPH (4 mg/kg) or AMPT/AMPH co-administration on NPY and MC4R expression on Day 1. Results showed Western Blots and the relative densitometric value for the protein products. Inserted photo showed the results of NPY, MC4R, and β-actin expression. Bars were mean ± SEM. *N* = 6 each group. *P < 0.05 vs. control. AMPT: α-methyl-para-tyrosine.

The change of food intake following AMPH treatment is shown in the upper panel of Figure 1. Statistical analysis by two-way ANOVA revealed significant dose-dependent [F(2,21) = 8.21, *p* < 0.05] and time-dependent effects [F(4,35) = 4.58, *p* < 0.05]. Followed by Dunnett’s test, it revealed that daily AMPH (2 mg/kg) produced marked decreases in food intake from Day1 to Day 2 (anorectic effect) and a gradual return to normal intake from Day 2 to Day 4 (tolerant effect), but daily AMPH (4 mg/kg) produced a continuous anorectic response during a 4-day period of time. Results showed in the lower panel of Figure 1 revealed that there was a significant difference between AMPH (4 mg/kg) group and AMPT + AMPH (4 mg/kg) group, indicating that AMPT could reverse AMPH-induced anorexia to normal level. Furthermore, the effect of AMPH (2 mg/kg) on Day 4 was significantly higher than that on Day 2, revealing that 2 mg/kg AMPH could induce gradually the tolerant effect. However, with a dose of 4 mg/kg AMPH, it could produce a continuous anorectic response during a 4-day period of drug treatment. The effect of AMPH on body weight change was in consistence with the alteration of feeding [8,40]. Based on these findings, AMPH (4 mg/kg) was employed for studies of AMPT/AMPH co-administration and Y1R antisense/AMPH co-administration since it could exert a greater anorectic effect which was more suitable than AMPH (2 mg/kg) for the examination of blocking effect of AMPT and Y1R antisense.

### Effects of AMPH on NPY, Y1R, c-Fos and c-Jun expression

**Figure 2:**
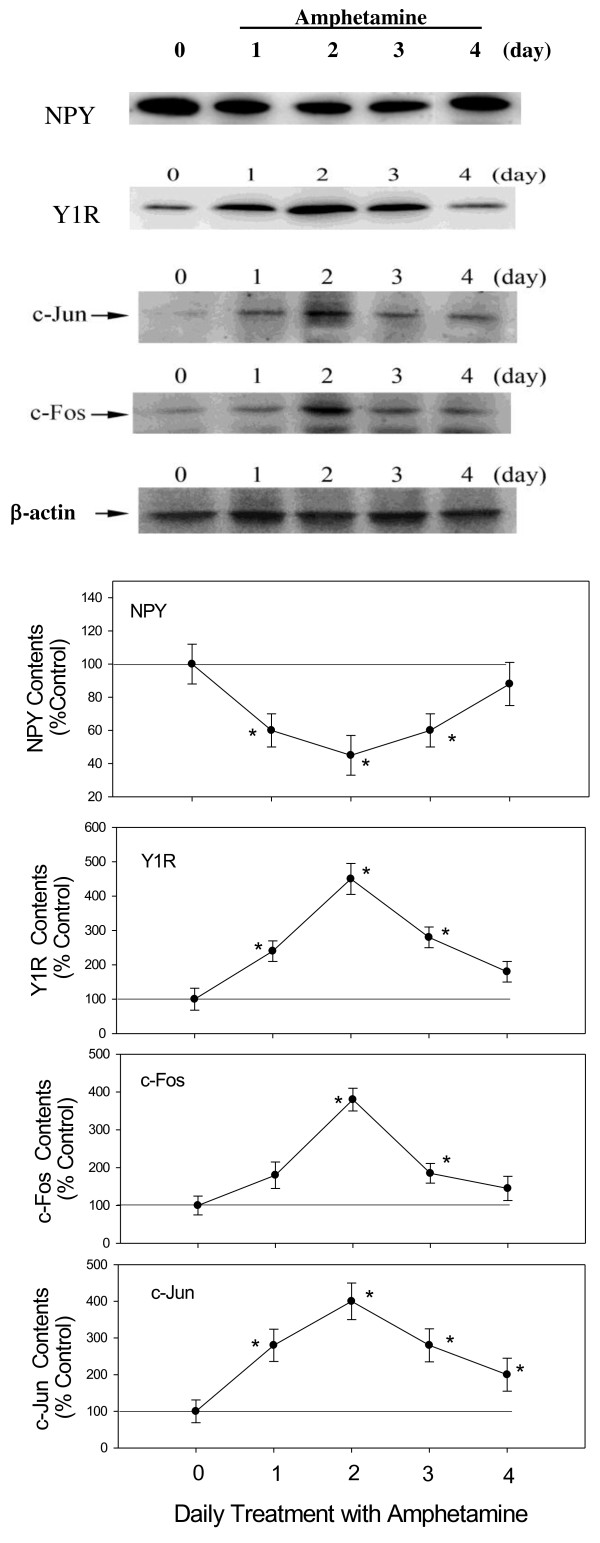
**Effects of daily AMPH on the expression of hypothalamic NPY, Y1R, c-Fos, and c-Jun over a 4-day period. ***Upper panel:* the results of Western Blot. *Lower panel:* relative densitometric values for Western Blot in control and 2 mg/kg AMPH-treated groups. Contents of protein in AMPH-treated groups were indicated as the percentage of the control group. Bars are the means ± SEM. *N* = 8 each group. * *p* < 0.05 vs. control. ^#^*p* < 0.05 vs. the AMPH-treated groups.

Results shown in Figure 2 revealed that daily AMPH resulted in a significant decrease of NPY during AMPH treatment, which was in accordance with the response of feeding response. However, daily 2 mg/kg AMPH resulted in the increases of c-Fos, c-Jun, and Y1R levels, which was expressed reciprocally to NPY level, compared to the control. Statistical analysis with one-way ANOVA indicated a decrease of NPY contents [F(4,35) = 5.74, *p* < 0.05] from Day 1 to Day 3 with a biggest decrease of about 55% on Day 2. However, it revealed a significant increase of c-Fos [F(4,35) = 7.12, *p* < 0.05], c-Jun [F(4,35) = 6.25, P < 0.05], and Y1R [F(4,35) = 4.57, *p* < 0.05] with a maximum increase of about 280%, 300% and 350%, respectively, on Day 2 compared to the control group. These results revealed that NPY was decreased and expressed in a manner reciprocal to that of Y1R, c-Fos, and c-Jun during AMPH treatment.

### The effect of AMPH on AP-1/DNA binding activity

**Figure 3:**
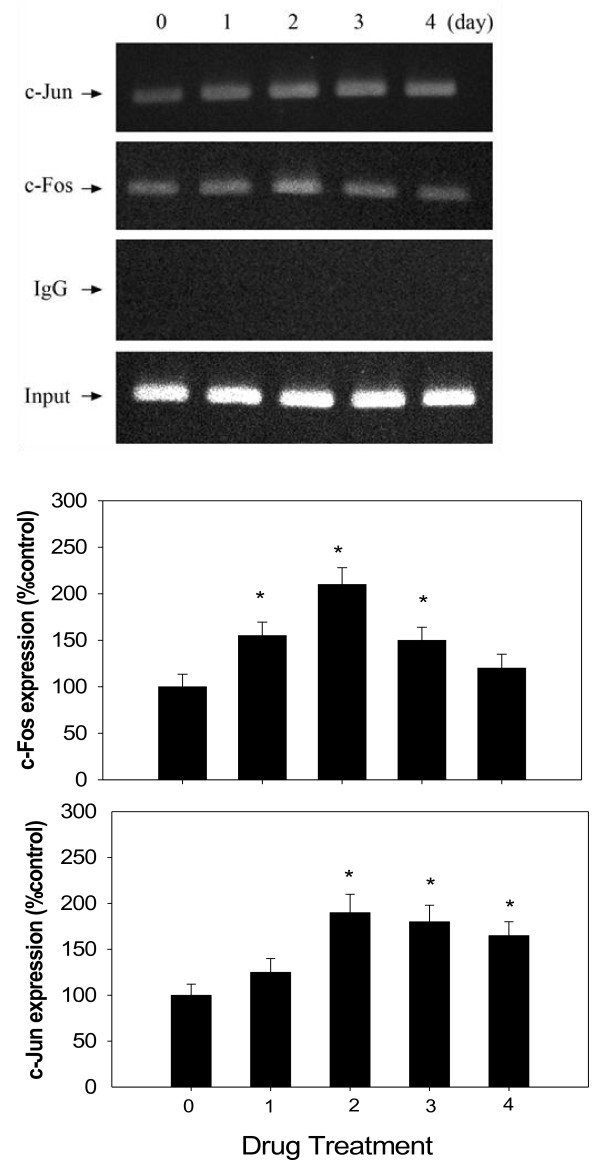
**The hypothalamus tissue was prepared for the ChIP assay using antibodies specific for c-Jun and c-Fos.** PCR amplification using primers designed against AP-1-binding sites was performed. Equal amounts of the soluble crosslinked chromatins present in each PCR were confirmed by the product for input. Rabbit polyclonal IgG was used as a negative control. Input, 1% of sonicated cross-linked chromatins. *Upper panel:* the result of ChIP analyzing AP-1/DNA binding activity. *Lower panel:* relative densitometric values for ChIP assay. Contents of AP-1/DNA binding activity were indicated as the percentage of the control group. Bars are the means ± SEM. *N* = 8 each group. * *p* < 0.05 vs. control.

Results shown in Figure 3 reveal that AMPH can increase AP-1 and DNA binding activity in the hypothalamus. Analysis with one-way ANOVA revealed the increases of c-Fos from Day 1 to Day 3 [*F*(4,25) = 2.68, *p* < 0.05] and c-Jun from Day 1 to Day 4 [*F*(4,25) = 2.98, *p* < 0.05] compared to the control. This result revealed that AP-1/DNA binding activity increased with the maximum response on Day 2 during AMPH treatment.

### The effect of ICV injections of Y1R antisense on AMPH anorexia

**Figure 4:**
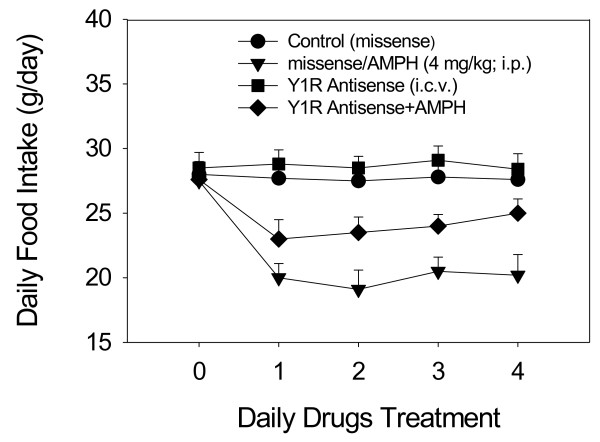
**The effect of Y1R antisense (or missense) pretreatment on daily AMPH-mediated food intake over a 4-day period.** Daily missense or antisense treatment (20 μg/10 μl/day, i.c.v.) was administered one hour before daily AMPH treatment. * *p* < 0.05 vs. the missense groups. ^#^*p* < 0.05 vs. the AMPH-treated groups of each treatment. Bars are the means ± SEM. *N* = 8 per group.

As shown in the lower panel of Figure 4, Y1R antisense alone-treated group could slightly but not significantly increase food intake from Day 1 to Day 4 compared to that in the control group. Moreover, Y1R antisense can partially reverse AMPH-induced anorexia, indicating the involvement of Y1R gene during AMPH treatment. Using two-way ANOVA to measure the effect of Y1R antisense, significant drug-dependent [F(3,28) = 5.88, *p* < 0.05] and time-dependent effects [F(4,35) = 6.35, *p* < 0.05] were revealed. Comparing the food intake between antisense/AMPH-treated and AMPH-treated rats, it revealed significant effects from Day 1 to Day 4. Furthermore, it also revealed significant effects from Day 1 to Day 4 if comparing between antisense/AMPH-treated and missense-treated (control) rats. The feeding response in missense-treated rats was similar to that in saline-treated rats. Moreover, the anorectic response in missense/AMPH-treated rats was not significantly changed when compared to that in AMPH-treated rats. These results revealed the noninterference of missense treatment in this study and also revealed that Y1R knockdown could modify the feeding responses in AMPH-treated rats.

### Effects of Y1R antisense on NPY, c-Fos, c-Jun, and Y1R expression

**Figure 5:**
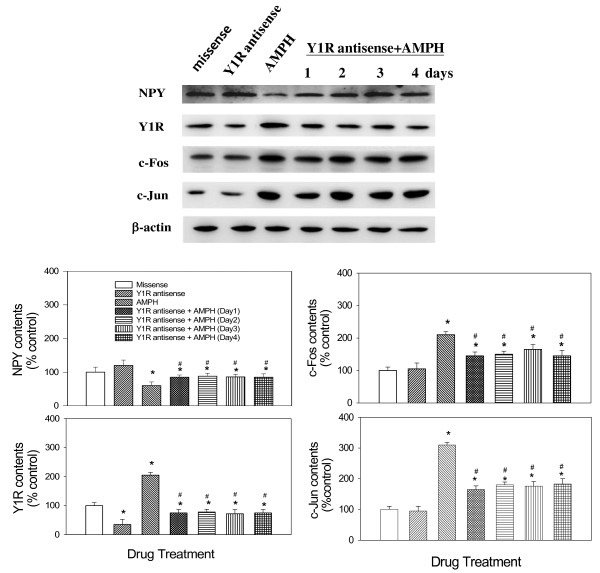
**The effect of Y1R antisense (or missense) pretreatment on AMPH-induced changes of NPY, Y1R, c-Fos, and c-Jun levels over a 4-day period.** Daily missense or antisense treatments (20 μg/10 μl/day, i.c.v.) were administered one hour before daily 4 mg/kg AMPH treatment. Results showed that Y1R antisense could partially restore c-Fos, and c-Jun levels toward normal. * *p* < 0.05 vs. the control (missense) groups of each treatment day. ^#^*p* < 0.05 vs. the AMPH-treated groups of each treatment day. Bars are the means ± SEM. *N* = 8 per group.

As shown in Figure 5, Y1R antisense by itself could reduce Y1R level compared to the control (missense-treated) group, revealing an efficient effect of Y1R antisense on Y1R expression. Moreover, a pretreatment with Y1R antisense in AMPH-treated rats resulted in partial restoration of NPY, Y1R, c-Fos, and c-Jun levels toward normal, revealing an involvement of Y1R in the regulation of NPY, c-Fos, and c-Jun contents. Using β-actin as the internal standard, the protein ratio of NPY, c-Fos, c-Jun, or Y1R over β-actin in each group was calculated and compared. By one-way ANOVA followed by Dunnett’s test, it revealed that NPY levels were decreased by about 43 ± 6% in AMPH-treated group, but increased about 15 ± 5% in antisense-treated group compared to the control groups [F(6,35) = 4.16, *p* < 0.05]. By contrast, Y1R levels were increased by about 100 ± 15% in AMPH-treated group but decreased by about 65 ± 10% in antisense-treated groups compared to the control group [F(6,35) = 5.52, *p* < 0.05]. Moreover, Y1R level showed significant effect in antisense/AMPH-treated group compared to AMPH-treated or antisense-treated group. Similarly, c-Fos and c-Jun contents were increased by about 105 ± 15% [F(6,35) = 4.85, *p* < 0.05] and 206 ± 16% [F(6,35) = 5.56, *p* < 0.05], respectively, in AMPH-treated group compared to the control group. Moreover, c-Fos, and c-Jun levels partially reversed to normal in antisense/AMPH-treated groups compared to AMPH-treated or antisense-treated group.

### Effects of BIBP-3226 pretreatment on feeding and changes of NPY, c-Fos, and c-Jun expression

**Figure 6:**
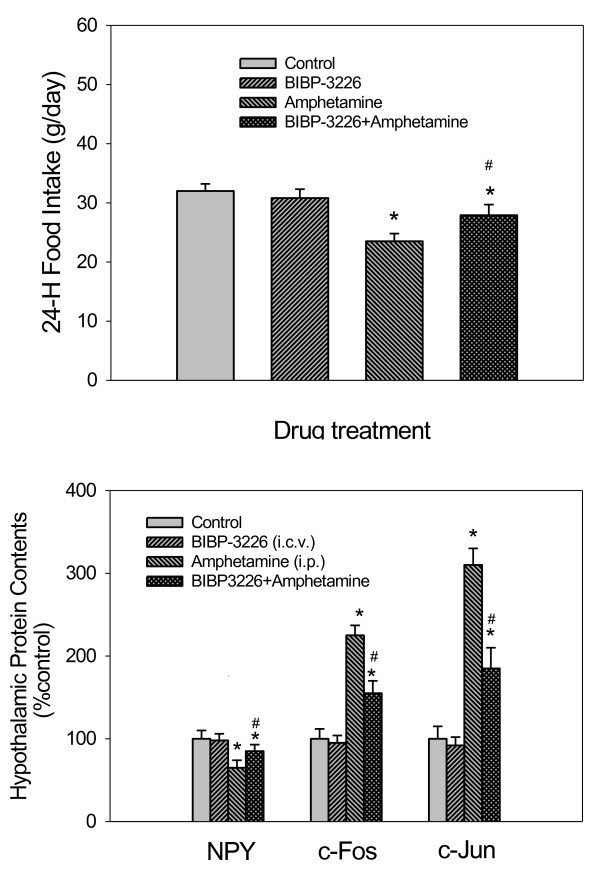
***Upper panel: *The effect of Y1R inhibitor (BIBP-3226) on 24-h AMPH-induced appetite suppression.** AMPH-induced appetite suppression could be modulated by prior administration of BIBP-3226 (80 nmol, i.c.v.). *Lower panel:* The effect of BIBP-3226 on the changes of hypothalamic NPY, c-Fos, and c-Jun in 4 mg/kg AMPH-treated rats during the first day of drug treatment. AMPH-induced changes of NPY, c-Fos, and c-Jun could be modulated by prior administration of BIBP-3226. Contents of NPY, c-Fos, and c-Jun were indicated as the percentage of controls. * *p* < 0.05 vs. the control (aCSF-treated, i.c.v.) group. ^#^*p* < 0.05 vs. the AMPH-treated groups. Bars are the means ± SEM. *N* = 8 per group.

As shown in the upper panel of Figure 6, it revealed that pretreatment with BIBP-3226 before 4 mg/kg AMPH could attenuate an AMPH-induced anorectic response. Statistical analysis with one-way ANOVA revealed a significant effect [F(3,28) = 7.42, *p* < 0.05]. AMPH could decrease the food intake by 50% compared to the control and pretreatment with BIBP-3226 before AMPH could reverse food intake by 50% compared to AMPH-treated group. The food intake in control (aCSF-treated) rats was similar to that in saline-treated rats, revealing the noninterference of vehicle in this study. Moreover, the expression of feeding in BIBP-3226-treated rats was slightly but not significantly reduced (decreased by 10% food intake) compared to that in vehicle-treated rats, revealing that BIBP-3226 had no significant effect on basal food intake in a 24-h testing period.

As shown in the lower panel of Figure 6, BIBP-3226 treatment alone didn’t affect the expression levels of NPY, c-Fos, and c-Jun compared to the control group. However, a pretreatment with BIBP-3226 in AMPH-treated rats resulted in partial restorations of NPY, c-Fos, and c-Jun levels toward normal level. Using β-actin as the internal standard, the protein ratio of NPY, c-Fos, and c-Jun over β-actin in each group was calculated and compared. By one-way ANOVA followed by Dunnett’s test (P < 0.05), it revealed that significant decrease of NPY content was observed in AMPH-treated and BIBP-3226/AMPH-treated groups compared to the control group [F(3,28) = 3.58, *p* < 0.05]. Moreover, BIBP-3226 could partially block NPY decrease about 52% compared to the AMPH-treated group. However, contents of c-Fos [F(3,28) = 4.52, *p* < 0.05], and c-Jun [F(3,28) = 5.11, *p* < 0.05] were increased in AMPH-treated group and BIBP-3226/AMPH-treated groups compared to the control group. Moreover, BIBP-3226 could partially block c-Fos, and c-Jun contents by about 50%, and 55%, respectively, compared to the AMPH-treated group.

## Discussion

Our current results have shown that cerebral CA participates in the control of NPY and MC4R expression. Moreover, both Y1R and AP-1 are involved in the regulation of AMPH-mediated appetite suppression and that they are increased and expressed in a pattern just opposite to the decrease of NPY during AMPH treatment. This happens because pretreatment with Y1R antisense (i.e., Y1R knockdown) or BIBP 3226 (a selective Y1R inhibitor) can modulate the expression of NPY, c-Fos, and c-Jun. These results show that Y1R play a functional role in regulating AP-1–mediated appetite control in AMPH-treated rats. These results expand our previous findings [26] and suggest that hypothalamic CA/NPY/Y1R/AP-1 signal pathway participates in the regulation of AMPH-induced anorexia.

Daily treatment with 2 mg/kg of AMPH decreased food intake and NPY expression during the initial two days of this study (an anorectic effect) and, in turn, reverse this effect gradually on the subsequent days, with food intake and NPY expression returning to normal (a tolerance to AMPH). Thus, hypothalamic NPY participated in both the anorectic response of AMPH, which was related to a decrease of NPY, and in the tolerant response of AMPH, which was related to NPY restoration [43]. Moreover, expression of Y1R, c-Fos, c-Jun, and AP-1 increased during AMPH treatment, with the maximum increase observed on Day 2. This manner of expression was just opposite to NPY expression, which showed the maximum decrease on Day 2. These results implied that Y1R, c-Fos, c-Jun, and AP-1 might function in a manner opposite that of NPY during the regulation of AMPH-evoked anorexia.

In the present study, both pretreatment with antisense to knock down Y1R expression or with antagonist to block Y1R activity could modulate the expression of NPY, c-Fos and c-Jun, indicating the involvement of Y1R in the regulation of NPY/AP1-mediated appetite suppression. This is in accordance with a previous report indicating that an intracerebral injection with a selective Y1R antagonist can inhibit c-Fos immunoreactivity in the area of the magnocellular paraventricular nucleus, which mediates the stimulation of NPY-induced feeding [44]. Thus, the possibility that the hypothalamic NPY-Y1R-AP1 signals played a role in the control of AMPH-mediated anorexia was considered.

Y1R expression increased during AMPH treatment. Although this increased expression was opposite to the decreased expression of NPY during AMPH treatment, it was consistent with the increased expression of POMC mRNA levels [8,11]. This result revealed that Y1R might play an essential role that is consistent with the function of the POMC neurons (an anorexigenic transmission) but is opposite to that of NPY neurons (an orexigenic transmission). Previous evidence has revealed that NPY can inhibit POMC-containing neurons via a unidirectional input from NPY to POMC [45,46]. Thus, the CA released during AMPH treatment might at first exert its inhibitory action on NPY neurons, which in turn increased (or disinhibited) POMC expression via the activation of Y1R.

POMC gene expression might be changed during AMPH treatment via Y1R/AP-1 signaling. Previously, we had examined the effect of NPY knockdown on NPY/Y1R/POMC signal pathway and found that NPY knockdown could enhance the increasing effects of Y1R and MC3R in AMPH-treated rats [8]. Recently, we have examined the effects of Y1R knockdown on NPY/Y1R/NF-κB/POMC signal pathway and found that Y1R knockdown reduce the increasing effects of Y1R, nuclear factor kappa B (NF-κB), and MC3R in AMPH-treated rats [47]. In the present study, Y1R knockdown could reduce anorectic response and NPY reduction, and reduce the increasing effects of Y1R and AP-1 in AMPH-treated rats. Thus, we suggest that NPY/Y1R/AP-1/POMC signal pathway is involved in regulating AMPH anorexia.

The increased expression of Y1R from Day 1 to Day 3 during AMPH treatment might be related to the activation of some transcription factors in POMC-containing neurons. The Y1R gene in rodents contains multiple regulatory elements, such as NF-κB, AP-1, and c-AMP response element–binding protein (CREB), which can be regulated by neuronal activity and may participate in the regulation of Y1R expression [48]. Thus, the expression of the Y1R gene in the hypothalamus may change during the regulation of energy balance, such as fasting, hypophagia, and diet-induced obesity [49]. In the present study, Y1R and AP-1 expression were increased during AMPH treatment and this increase was just opposite to the decrease of NPY, revealing the involvement of NPY-Y1R-AP1 signaling in the regulation of AMPH-induced anorexia. Our previous studies revealed that both CREB [50] and NF-κB [8] genes in POMC-containing neurons were up-regulated and expressed in a manner similar to that of the Y1R gene during a 4-day period of AMPH treatment. Recently, we found that Y1R was involved in regulating CREB [51] and NF-κB [47] expression in AMPH or PPA-treated rats, revealing the activation of Y1R/CREB and Y1R/NF-κB signals during AMPH (or PPA) treatment. These results implied that the activation of Y1R-AP1 signaling, perhaps together with the co-activation of Y1R-CREB and Y1R-NFκB signals, might function together in the modulation of POMC gene expression during AMPH treatment. Moreover, the co-activation of Y1R/AP-1, Y1R/CREB, and Y1R/NFκB signals during AMPH treatment might also explain why the pretreatment with Y1R antisense or BIBP-3226 partially blocked the effects of AMPH on c-Fos and c-Jun levels in the present study.

The co-activation of Y1R and AP-1 during AMPH treatment might be involved in the regulation of oxidative stress in the brain. Our previous reports revealed that several anti-oxidative enzymes, such as superoxide dismutase [26] and glutathione peroxidase [10], are elevated and expressed similar to Y1R and AP-1 expression, which was seen in the present study during the 4-day AMPH treatment period. Moreover, brain NPY is associated with the anti-stress response [52], and brain Y1R can be modulated by different kinds of brain insults, such as stress and seizure activity [48,53]. Furthermore, AP-1 can be rapidly induced by brain injury or drug treatment [54,55] and rats treated with methAMPH might cause prolonged increase of AP-1 because oxygen-based free radicals are known activators of AP-1 [56]. Thus, NPY-Y1R-AP1 signal transduction in the brain might play a functional role in anti-oxidative stress in AMPH-treated rats. As the stress hormone glucocorticoid can modulate both Y1R [57] and AP-1 [58,59] in the brain, we suggested that the release of endogenous NPY and the activation of Y1R and AP-1 in the nervous system might be one of the essential routes to activate anti-stress system, such as the activations of POMC, glucocorticoid, and anti-oxidative enzymes, during stress exposure in the brain.

Decreased expression of NPY-AP1 system in the hypothalamus might be involved in higher levels of anorexia, while decreased expression of NPY-AP1 system in the amygdale might involve higher levels of anxiety. In the amygdale, evidence has shown that decreased expression of the NPY gene is related to the increased anxiety and alcohol intake [15] and that c-Fos immunoreactivity is increased after the administration of anxiogenic drugs [60,61]. Moreover, dopamine plays an important role in fear and anxiety by modulating the anxiogenic output of the amygdale [62,63]. Thus, rats in a feeding state of anorexia were found to be similar to those in a mental state of anxiety because both animals were in a state of decreased NPY and increased AP-1 signaling. This could explain why AMPH could induce both anxiety-related effects [64] and anorectic responses [21] and why c-Fos activation can regulate anxiety [65] and modulate anorexia [26] in AMPH-treated animals.

Although we didn’t detect the expression of NPY receptor 2 (NPY2R), its’ possible role in the regulation of AMPH-induced anorexia should be considered. The deletion of NPY2R in the adult mouse hypothalamus leads to transiently decreased body weight and increased food intake, indicating the functional role of the hypothalamic NPY2R in controlling feeding behavior [66,67]. A recent publication [68] has suggested that in chromaffin cells, which are modified neuroendocrine cells all expressing NPY and sharing some commons with hypothalamic NPY/AgRP neurons, NPY expression is negatively regulated by NPY2R but not NPY1R or NPY5R.

Drugs that target for NPY receptors have been developed as potential anti-obesity drugs [49,69]. Although potent and selective antagonists of Y1R and Y5R have been developed [49,70], mechanisms for signal transduction downstream to Y1R and Y5R are not clear. The present study provides evidence that the activation of NPY/Y1R/AP-1 signaling in the hypothalamus might help regulate the anorectic response of AMPH.

## Conclusion

The present results suggest that cerebral CA is involved in controlling hypothalamic NPY and MC4R expression and that hypothalamic Y1R participates in regulating NPY/AP-1–mediated appetite suppression. These results may further the understanding of the role of molecular mechanisms in the appetite-suppressing effect of AMPH.

## Abbreviations

aCSF: Artificial cerebrospinal fluid; ANOVA: Analysis of variance; AMPH: Amphetamine; AP-1: Activator protein-1; CREB: c-AMP response element–binding protein; CA: Catecholamine; ChIP: Chromatin immunoprecipitation; DAG: Diacylglycerol; IEG: Immediate early genes; i.c.v.: Intracerebroventricular; MC4R: Melanocortin receptor 4; NPY: Neuropeptide Y; IP: Intraperitoneally; NF-κB: Nuclear factor kappa B; ODN: Oligodeoxynucleotides; PPA: Phenylpropanolamine; POMC: Pro-opiomelanocortin; S-ODN: Phosphorothioate oligodeoxynucleotides; Y1R: NPY-Y1 receptor; Y5R: NPY-Y5 receptor.

## Competing interests

The authors declare that they have no competing interest.

## Authors’ contribution

Study concept and design, and interpretation of the results were performed by all authors. Kuo DY had full access to all the data in the study and takes responsibility for all aspects of the study including integrity of data accuracy and data analysis. All authors read and approved the final manuscript.
